# Parent emotion regulation difficulties statistically mediate the association between parental adverse childhood experiences and child emotion regulation

**DOI:** 10.3389/fgwh.2025.1587786

**Published:** 2025-11-28

**Authors:** Sarah E. Freeman, Angela D. Staples, Tamara M. Loverich, Madison Hannapel, Jamie M. Lawler

**Affiliations:** Department of Psychology, Eastern Michigan University, Ypsilanti, MI, United States

**Keywords:** intergenerational transmission, adverse childhood experiences, emotion regulation, emotion related socialization behaviors, parenting

## Abstract

**Introduction:**

Emotion regulation (ER) abilities predict positive outcomes among children. While parenting behaviors that promote young children's ER development have been widely studied, less is known about how a parent's history of early trauma may interfere with their use of effective strategies, despite well-documented next-generation impacts of early adversity. The present study evaluates the statistical mediating role of parent ER and parent emotion-related socialization behaviors (ERSBs) in the relation between parent adverse childhood experiences (ACEs) and child ER.

**Methods:**

Data come from a cross-sectional study of 214 caregivers of children ages 2 through 5 (inclusive) participating in a larger online study examining parenting factors that are associated with children's self-regulation development. Measures used include Traditional and Expanded ACEs Scales, the Difficulties in Emotion Regulation Scale Short Form, the Coping with Toddlers' Negative Emotions Scale, and the Emotion Regulation Checklist. Data analysis involved correlation and mediation analyses.

**Results:**

Parent *difficulties* in ER statistically mediated the association between parent ACEs and child ER such that a higher expanded ACEs score was associated with more parent difficulties in ER, and these difficulties were related to lower child ER. Although parent ER and emotion-accepting ERSBs independently contribute to child ER, data did not support a statistical mediational role for ERSBs or multiple statistical mediation.

**Discussion:**

Study findings implicate parent ER as a potential target for parenting interventions aiming to promote child ER among parents with a history of adversity, suggesting that support for parent ER may be one avenue for the reduction of intergenerational transmission of trauma.

## Introduction

Although there is solid evidence in the literature regarding the negative impact of parental adverse childhood experiences (ACEs) on subsequent generations (1), the specific mechanisms associated with this intergenerational transmission are not clear. Reviews examining possible mechanisms have implicated parent mental health difficulties (e.g., depression and anxiety) as possible mediators accounting for next-generation emotional difficulties, but specific “positive” or “negative” parenting practices have not emerged as clear mechanisms for the intergenerational transmission of trauma (2). Emotion regulatory abilities set the stage for both the long-term and short-term socio-emotional success of young children, making emotion regulation (ER) a foundational construct in the field of child development. Despite being well-established that parenting factors play a role in shaping children's ER development (3, 4), and that ACEs can influence many factors related to parenting (5–7), little research has connected the dots between these relations to investigate the mediating roles both of parent ER and parent emotion socialization in explaining the relation between parent ACEs on child ER. This study addresses this gap in the literature by examining whether these constructs statistically mediate this association between parent ACEs and child ER.

Emotion regulation can be conceived as the intentional or automatic manipulation of one's own emotional experiences and expressions in order to achieve a desired goal or outcome (8). According to Thompson (9), such a goal may include controlling the occurrence, duration, intensity, or expression of an emotion. Children demonstrating more adaptive emotion regulation are consistently found to exhibit fewer internalizing and externalizing symptoms in both clinically referred and non-clinically referred samples (10, 11). ER has also been found to predict academic preparedness (12) and success (13), as well as improved social functioning (14, 15). Additionally, young children with more advanced ER skills may also perceive themselves to be both more socially accepted by peers and more competent (16), suggesting that ER has implications for children's burgeoning self-esteem. The social impacts of *poor* ER may also have significant, long-term repercussions. In a longitudinal study, young children's observed emotion dysregulation during preschool significantly predicted peer rejection in middle childhood, which subsequently predicted their antisocial behavior reported by teachers in early adolescence (17). Emotion dysregulation has consistently been linked with later psychopathology and health problems, including anxiety, aggression, and eating pathology (18, 19), alcohol-related problems (20), and the development of PTSD following trauma exposure (21).

### Development of emotion regulation

Given that children's development of ER skills represents a critical foundational milestone with lifelong implications, it is important to understand how these skills develop, as well as what factors aid or hinder their promotion. Several models have been proposed to help researchers investigate the role that parents and the broader family context play in helping to socialize children's emotions and shape their ER capacities, including Eisenberg's (22) model of the socialization of emotion and the Tripartite Model of the Impact of the Family on Children's Emotion Regulation and Adjustment (23). Efforts on the part of parents to help their children understand and regulate their emotions have been referred to in the literature as “emotion-related socialization behaviors” (ERSBs), a key construct in Eisenberg and colleagues' (22) model of the socialization of emotion. According to this model, a caregiver's ERSBs may directly influence child emotion-related outcomes, including how they understand, experience, express, and regulate emotions, and they may also influence child outcomes indirectly via their influence on children's emotional arousal (22). While parents' ERSBs are the focal point of this model, these behaviors do not exist in a vacuum. Thus, Eisenberg's model identifies key predictors and potential moderators of the relation between ERSBs and child emotion-related outcomes, including individual parent characteristics.

The Tripartite Model (23) bears significant resemblance to Eisenberg's model but focuses on the roles of three specific factors that influence children's ER development: observation of parents' emotions and ER, specific parenting practices that caregivers engage in related to emotions and ER, and the emotional climate of the family (23). Like in Eisenberg's model, each of these parenting and familial factors can be influenced by individual parent characteristics, which may include considerations such as parents' own ER, mental health, and caregiving history (23). Thus, what these models share is an emphasis on the role of specific parenting behaviors and practices in the socialization of young children's emotions and ER while acknowledging the role that individual caregiver characteristics may play in influencing their ERSBs.

#### Evidence of the effects of parent ER and ERSBs on children's ER

One way parents teach their children about emotions and emotion regulation is through modeling. That is, by observing how their caregivers express and manage their own emotions (or do not, as the case may be), children develop expectations and beliefs about emotions and ER, which influence how they express and attempt to regulate their own emotions (3, 4). The literature base examining relations between parent emotion regulation and child emotion regulation consistently supports the presence of a positive association between parent ER and child ER (24–28). For example, in their study including over 400 mothers and their young children between ages three and seven, Crespo and colleagues (25) reported a significant positive association between maternal ER difficulties and child ER difficulties (*r* = .22) and between maternal ER difficulties and child emotion lability/negativity (*r* = .37). In their sample, child ER difficulties were further found to statistically mediate the relation between parent ER difficulties and children's behavior problems, highlighting additional important implications of the association between parent ER and child ER. A recent longitudinal study utilizing cross-lagged path analyses found evidence that Dutch mothers' self-reported emotion regulation difficulties at 6 months and 1.5 years postpartum significantly predicted their children's socio-emotional problems at ages 1 and 3 years, respectively. Interestingly, child socio-emotional problems at 1 year significantly predicted maternal ER difficulties at 1.5 years postpartum, suggesting that these pathways may be bidirectional in nature (28).

Furthermore, parents' use of emotion-accepting emotion socializing behaviors, often collectively called “supportive” strategies, is positively associated with children's ER (14, 29). How parents respond to their children's emotional displays, and in particular, to those that are negative in valence, influences how children evaluate and accept their own emotions (3, 4). For example, in a sample of maltreating mothers, maternal emotional support mediated the relation between maltreatment and children's emotional expression (30). Furthermore, mothers' emotional expressivity, a component of emotion socialization, is linked to children's emotional regulatory abilities, such that positive expressivity is associated with greater emotion regulation capacity, while maternal negative expressivity is associated with lower ER (14).

While there is consistent support for a link between parents' ER and children's ER, and between emotion socialization practices and children's ER, it is unclear whether emotion socialization plays a mediating role between parent ER and child ER. One study failed to find support for emotion socialization as a mediator, instead finding that emotion socialization and parent emotion regulation had independent effects on children's ER (24). However, another study found partial support for the mediational role of emotion socialization on this relation (26). Specifically, emotion-avoiding emotion socializing behaviors, often referred to as “unsupportive” emotional parenting, statistically mediated the link between maternal dysregulation and child *dysregulation*, but not the relation between maternal dysregulation and child regulation. Overall, data suggest that there may be a stronger relation between parent's emotion *dysregulation* and emotion-*avoiding* emotion socialization behaviors than between parent's emotion regulation and emotion-accepting emotion socialization behaviors (26, 31).

### Parent ACEs

Individual parenting factors and experiences may limit or promote a caregiver's ability to support children's ER development. Although limited studies have evaluated the relation between parent ACEs specifically and child ER, the extant literature suggests that parent ACEs are associated with poorer ER outcomes among children (32, 33) and multiple studies have linked parental ACEs with increased offspring internalizing and externalizing problems (1, 34), and mental health problems broadly (34) which are associated with ER difficulties. More prevalent is the literature linking maternal history of child maltreatment with child regulatory outcomes. For example, DeOliveira and colleagues (35) found that mothers' experiences of physical and emotional abuse were associated with poor child ER capacities during a frustration task. Similarly, in a longitudinal study examining the effects of maternal childhood maltreatment on their offspring's regulatory abilities during preadolescence, maltreatment history was found to predict regulatory abilities via maternal controlling parenting behaviors (36).

Given the mounting evidence pointing toward an association between parental ACEs and related constructs and diminished child ER abilities, it is important to consider parenting variables that may account for this relation. As discussed, many parent and emotion-related parenting variables, including parent ER and ERSBs have been found to relate to child ER outcomes. It is important to consider how these parenting variables may relate to a caregiver's history of early adversity.

ER difficulties have been implicated as a mediator accounting for the well-documented associations between ACEs and myriad negative physical and mental health outcomes, including psychological distress (37); depression, PTSD, and physical wellbeing (38); interpersonal difficulties (39); and anxiety (40). Thus, not only has adversity during childhood been consistently linked to ER difficulties in adulthood, but it is these particular difficulties that may account for the negative physical and mental health outcomes individuals with ACEs are more likely to experience in adulthood.

As discussed, past studies have linked parents' ER and ERSBs, particularly when examining the association between parental emotional *dysregulation* and the use of *unsupportive* emotion parenting practices (26, 31). However, the existing body of research has not yet demonstrated an association between ACEs and ERSBs. The available literature focuses instead on the connection between a related construct, parents' childhood maltreatment, and ERSBs. For example, DeOliveira and colleagues (35) found that mothers with a history of physical and emotional abuse responded to their 4- to 6-year-old children with more hostility and less emotional availability than mothers without such a history. Furthermore, mothers with a history of abuse were more likely to misinterpret infants' emotions (35). Likewise, Rea and Schaffer (41) found significant negative correlations between each type of childhood abuse and neglect measured and parent-reported emotionally supportive parenting behaviors, although maltreatment history was not significantly related to parent-reported *unsupportive* parenting behaviors.

Another study tested a serial mediation model evaluating the effects of parent polyvictimization (i.e., sexual abuse, physical abuse, emotional maltreatment, and neglect) on 8 to 12-year-old children's emotion inhibition through parental ER and parents' unsupportive ERSBs (42). In this study, authors found support for the full sequential mediational model, and results further supported a significant indirect effect of polyvictimization on emotion inhibition via unsupportive emotion socialization (i.e., independent of the effects of parent ER).

### The present study

**Figure 1:**
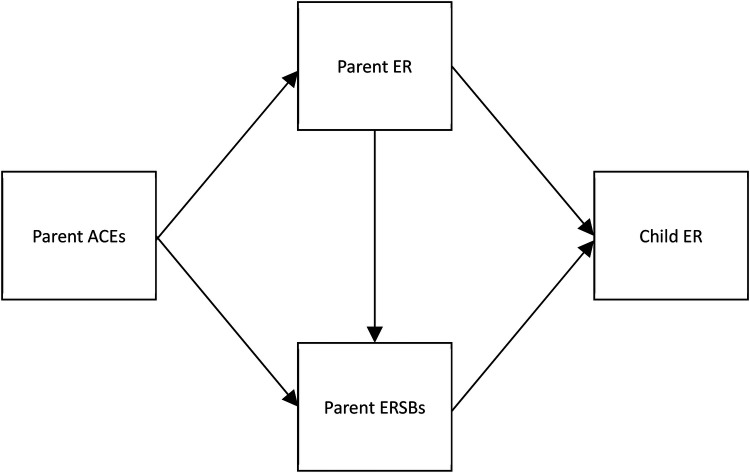
Hypothesized conceptual model showing parent emotion regulation and parent emotion-related socialization behaviors as statistical mediators of the association between parental adverse childhood experiences and child emotion regulation. ACEs, adverse childhood experiences; ER, emotion regulation; ERSBs, emotion-related socialization behaviors.

While parenting and family variables that contribute to children's ER development have been widely studied, less is known about how potential risk factors, such as a parent's history of adversity during childhood, may operate to compromise parents' ability to promote the development of ER among young children. The purpose of this study is to address these gaps in the literature by testing two possible constructs that may statistically mediate the association between parental ACEs and child ER: parent ER and parent ERSBs (conceptual model presented in Figure 1). If there is indeed evidence of statistical indirect effects of these constructs on the relation between parents' early adversity and child ER, it is important to distinguish the specific role of each construct in this pathway. The study's hypotheses are as follows: (1a) Parent *difficulties* in ER and parent *emotion-avoiding* ERSBs will statistically mediate the association between parent ACEs and child ER such that higher ACEs will be associated with more *difficulties* in parent ER, which will be positively associated with parent *emotion-avoiding* ERSBs, which will be negatively related to child ER, and (1b) Parent *difficulties* in ER and parent *emotion-accepting* ERSBs will statistically mediate the association between parent ACEs and child ER such that higher ACEs will be associated with more *difficulties* in parent ER, which will be negatively associated with parent *emotion-accepting* ERSBs, which will be related to lower child ER. This study will also examine the possible statistical indirect effects of each construct independently. Hypotheses regarding simple statistical mediation are as follows: (2) Parent *difficulties* in ER will statistically mediate the association between parent ACEs and child ER; (3a) Parent *emotion-avoiding* ERSBs will statistically mediate the association between parent ACEs and child ER; (3b) Parent *emotion-accepting* ERSBs will statistically mediate the association between parent ACEs and child ER; (4a) Parent *difficulties* in ER will statistically mediate the association between parent ACEs and parent *emotion-avoiding* ERSBs; (4b) Parent *difficulties* in ER will statistically mediate the association between parent ACEs and parent *emotion-accepting* ERSBs; (5a) Parent *emotion-avoiding* ERSBs will statistically mediate the association between parent ER and child ER; (5b) Parent *emotion-accepting* ERSBs will statistically mediate the association between parent ACEs and child ER. Clarity regarding the nature of each construct's role can improve precision and goal-identification among interventions targeting child ER development.

Additionally, while many studies examining the intergenerational effects of early adversity have focused specifically on childhood maltreatment as a conceptualization of early adversity, the present study measures early adversity using a broader framework that includes these forms of maltreatment in addition to other indices of household dysfunction, the Adverse Childhood Experiences scale (ACEs; 43), as well as neighborhood and community-based adversity (the Expanded Adverse Childhood Experiences Scale; 44). The use of more inclusive measures of early adversity allows for the possibility of understanding whether and how cumulative adversity taking multiple forms may be associated with disruptions in this pathway.

## Methods

### Participants

The present study utilized self-report data from a larger, cross-sectional, online study designed to evaluate parenting and family factors that are associated with children's development of self-regulation skills. Informed consent was obtained online from all individual participants included in the study directly prior to survey administration. Caregivers were eligible for participation in this study based on their age (i.e., they were at least 18), having at least one child between two and five (inclusive), presently living in the United States or the United Kingdom, and socioeconomic risk (i.e., having at one point reported a self-rated score of 5 or lower on a 10-point subjective socioeconomic status scale). Participants were recruited via Prolific Academic, an online participant recruitment service, and all questionnaires were administered via an online survey that took about 60–75 min. Permission to conduct this research was obtained from the University Human Subjects Review Committee of the administering institution.

Data were collected from 300 caregivers. Of the 300 caregivers who began the study, 76 respondents were excluded from completing the full survey automatically due to inconsistent responding or not having a child in the correct age range. Of the 224 participants remaining, there were 214 cases with complete data that were included in the study. Due to the very small number (*n* = 10) of cases with missingness that could not be explained by failing a consistency check, patterns of missingness could not be further probed using *t*-tests.

Because data came from a sample that included participants residing in two different countries, and national and cultural factors may influence parenting styles, independent sample *t*-tests were used to examine between-group differences in key study variables based on whether participants reported being located in the United States or the United Kingdom. No significant between-group differences were identified (all *p* values > .05). Because there were no significant differences between groups, the entire sample was analyzed together.

The majority of the caregivers (72.0%) identified as female and their average age was 33.5 years. Target children of caregivers who participated in the study were 58.0% male (*n* = 124) and the average age was 3.9 years. Parents in the study experienced on average 3 traditional ACEs and 5.3 expanded ACEs. The most commonly reported ACEs endorsed were emotional abuse (53.3%), living with a caregiver who was mentally ill (43.9%), physical abuse (40.8%), and parental separation (40.8%). Thirty-nine percent of the sample (*n* = 84) reported experiencing four or more traditional ACEs. In terms of socioeconomic status, parents' average self-rated SES was 4.42 (out of 10) at the time of the study. See Table 1 for a full breakdown of demographic characteristics of the sample.

**Table 1:** Demographic characteristics of the sample population.

Characteristic	Parent	Child
	% (*n*)	% (*n*)
Employment		
Full	42.5 (91)	
Part	25.2 (54)	
Not working	32.2 (69)	
Marital status		
Married	52.3 (112)	
Unmarried	47.7 (102)	
Race		
White	82.2 (176)	78.0 (167)
Black/African American	10.7 (23)	10.3 (22)
Asian	3.3 (7)	3.3 (7)
White and Black/African American	0.9 (2)	2.8 (6)
White and American Indian or Alaska Native	0.5 (1)	1.4 (3)
White and Native Hawaiian or Pacific Islander	0.5 (1)	0.0 (0)
White and Other	0.5 (1)	0.5 (1)
White and Asian	0.0 (0)	0.9 (2)
Other	1.4 (3)	2.8 (6)
Education		
<High school degree	0.9 (2)	
High school degree	23.8 (51)	
GED	0.5 (1)	
Some college	22.4 (48)	
Associate's degree	10.7 (23)	
Bachelor's degree	26.2 (56)	
Master's degree	13.6 (28)	
Professional degree	0.9 (2)	
Doctoral degree	1.4 (3)	

*n* = 214.

### Measures

#### Demographic questionnaire

Demographic and eligibility questions were included to enable description of demographic characteristics of participants who enrolled and to identify potential covariates that may be relevant for analyses. Sociodemographic variables included participant race, age, gender, education level, employment status, household income, marital status, and self-rated socioeconomic standing. Self-rated socioeconomic standing was evaluated twice—once, when participants initially signed up for Prolific, and again when they began the study. Although participants were only invited to complete the study if they originally endorsed a score of 5 or lower (out of 10), participants were not excluded from participating if their self-rating was higher than 5 at the time of the study. Child demographic variables included age, race, and gender.

#### Adverse childhood experiences

The traditional ACEs questionnaire (43) is a 10-item measure that retrospectively assesses whether an individual experienced certain forms of adversity during the first 18 years of their life. Items include questions regarding experiences of physical, emotional, or sexual abuse; physical or emotional neglect; and household dysfunction (e.g., witnessing domestic violence, having parents who were separated or divorced, or living with a household member who misused substances, was mentally ill, or was incarcerated). Traditional ACE scores range from 0 to 10, with 0 representing no exposure to any of the forms of adversity identified in the measure, and 10 representing exposure to all 10 experiences. This scale has good internal consistency (Cronbach's *α* = .88; 45) and good test-retest reliability for individual items and overall score (46).

The expanded version of the ACEs questionnaire (44) includes additional questions related to adversity that may occur outside of the home context, including experiencing racism, witnessing violence, living in an unsafe neighborhood, experiencing bullying, and living in a foster home during the first 18 years of life. Furthermore, the expanded ACEs scale excludes experiencing parent divorce or separation. The use of expanded ACEs enables researchers to capture a broader range of experiences that may influence the health and wellbeing of diverse populations and paints a clearer picture of true experiences of early adversity (44). For the purposes of the present study, each individual question was scored as present or absent based on criteria laid out by Cronholm and colleagues (44), such that scores could range from 0 to 21.

#### Difficulties in emotion regulation scale short form (DERS-SF)

The DERS-SF (47) is an 18-item self-report measure adapted from the original, well-validated 36-item DERS (48). The DERS-SF is used to evaluate ER problems in adolescents and adults and consists of six subscales, including nonacceptance of emotional responses, difficulties engaging in goal-directed behavior, impulse control difficulties, lack of emotional awareness, limited access to emotion regulation strategies, and lack of emotional clarity. A total difficulties score was calculated and used in the present study by combining subscale scores. Internal consistency across subscales for the DERS-SF is good (Cronbach's *α* ranges from .78 to .91), subscales correlate highly with the original 36-item DERS subscales (ranges from .90 to .97), and concurrent validity of the DERS-SF is comparable to the original DERS (47).

#### Coping with toddlers' negative emotions scale (CTNES)

The CTNES (49) was adapted from the Coping with Children's Negative Emotions Scale (CCNES; 50). Utilizing a series of 12 hypothetical situations that describe a toddler's negative (i.e., upset, angry, or distressed) reactions to a situation, the CTNES asks parents to rate how likely they would be to react in certain ways. A thirteenth item was added to this scale in the present study in order to capture an additional scenario characterizing typical toddler behaviors that may occur frequently and was not otherwise included in the measure. Each scenario provides seven possible reactions, which parents rate on a 7-point scale from “very unlikely” to “very likely” to be their reaction. The CTNES consists of seven subscales including distress reactions, punitive reactions, minimizing reactions, expressive encouragement, emotion focused reactions, problem focused reactions, and granting the child's wish (49). Internal consistency ranges from good to excellent across most subscales (Cronbach's *α* ranges from .75 to .93) with the exception of the “granting the child's wish” subscale, which is acceptable (Cronbach's *α* = .67). Test-retest reliability of scales is good (*r*s range from .65 to .81; 45). A principal component factor analysis conducted by the scale's developers identified the punitive reactions and minimizing reactions subscales as belonging to a common emotion-avoiding factor (termed “unsupportive strategies”), while problem-focused, emotion-focused, and expressive encouragement subscales belonged to a common emotion-accepting factor (termed “supportive strategies”; 49). As such, total emotion-accepting and emotion-avoiding emotion socialization scores were calculated by summing scores from each factor's respective subscales.

#### Emotion regulation checklist (ERC)

The ERC (51) is a 24-item measure of children's ER designed to be completed by a parent or other adult. This measure assesses children's affective lability, intensity, valence, flexibility, and situational appropriateness, and includes two subscales: Lability/Negativity and Emotion Regulation. The Lability/Negativity subscale includes items indicating poor ER capacity, and the Emotion Regulation subscale includes items indicating the presence of adaptive ER strategies and a lack of flat affect. To capture overall effectiveness in utilizing ER strategies, a total ER score was calculated by combining the ER subscale score with the reverse-scored Lability/Negativity subscale score. Internal consistency is excellent for the Lability/Negativity subscale (Cronbach's *α* = .96) and good for the Emotion Regulation subscale (Cronbach's *α* = .83; 51).

## Results

### Bivariate correlations

To understand relations between key study variables and identify demographic covariates, bivariate correlations were run. See Table 2 for means, standard deviations, and correlations between key study variables. Although expanded and traditional ACEs were highly correlated (*r* = .94, *p* < .01), correlations between key study variables and both ACEs measures are reported since findings varied slightly depending on the measure used.

**Table 2:** Means, standard deviations, and bivariate correlations of key study variables.

Variable	*M* (SD)	Range (possible)	1	2	3	4	5	6	7	8
Traditional ACEs	3.00 (2.51)	0–10 (0–10)								
Expanded ACES	5.32 (4.44)	0–20 (0–21)	.94***							
Child ER	6.38 (0.69)	4.45–7.80 (2–8)	−.07	−.12*						
Emotion-avoiding ERSBs	5.48 (2.16)	2.08–11.67 (2–14)	−.12*	−.06	−.10					
Emotion-accepting ERSBs	17.02 (2.55)	7.25–21.00 (3–21)	.09	.03	.37***	−.33***				
Parent ER	15.16 (4.03)	7.33–29.33 (6–30)	.25***	.22***	−.21***	.05	.11			
Parent age	33.52 (6.06)	20–51 (18+)	−.05	−.04	.04	−.19***	.06	−.19***		
SES	(4.42 (1.24)	1–9 (1–10)	−.23***	−.24***	.15**	.10	−.02	−.14**	.02	
Parent gender	0.29 (0.48)		−.06	.01	−.04	0.24***	−0.26***	−0.02	.10	−.01

ACEs, adverse childhood experiences; ER, emotion regulation; ERSBs, emotion-related socialization behaviors; SES, socioeconomic status. For parent gender, 0 = female, 1 = male; nonbinary parent (*n* = 1) excluded from correlations including gender.

* *p* < .10.

** *p* < .05.

*** *p* < .01.

#### Emotion-related parenting variables and children's ER

Parent *difficulties* in ER were found to be negatively correlated with child ER such that greater parental difficulty with ER was associated with poorer next-generaion ER outcomes (*r* = −.21, *p* < .01). While no significant correlation was found between parent *emotion-avoiding* ERSBs and child ER, parent *emotion-accepting* ERSBs were found to be positively correlated with children's ER, such that higher endorsement of more accepting emotion-related parenting practices was associated with better child ER (*r* = .37, *p* < .01). No significant associations were found between parent's ER difficulties and either emotion-avoiding or emotion-accepting ERSBs.

#### Associations with parent ACEs

Although traditional ACEs were not found to be associated with child ER, there was a small, nonsignificant negative correlation between expanded ACEs and child ER (*r* = −.12, *p* = .09). Furthermore, parent ACEs were positively associated with parent *difficulties* in ER for both traditional (*r* = .25, *p* < .01) and expanded (*r* = .22, *p* < .01) ACEs. In contrast, there was no correlation found between parent ACEs (expanded or traditional) and *emotion-accepting* ERSBs. Analyses also revealed a small, nonsignificant negative correlation between parent traditional ACEs only and *emotion-avoiding* ERSBs (*r* = −.12, *p* = .09).

#### Demographic covariates

Bivariate analyses between demographic variables and key study variables revealed significant associations between study variables of interest and parent age, self-reported socioeconomic status, and parent gender (see Table 2). Given associations between key study variables and parent age, self-reported SES, and parent gender, these variables were included as covariates in statistical mediation analyses.

### Multiple statistical mediation analyses

A primary aim of this study was to test a multiple statistical mediation model in which parent ER and ERSBs statistically mediate the relation between parent ACEs and child ER. All mediational analyses were conducted using the R version of PROCESS (52), and bootstrapped 95% confidence intervals (5,000 iterations) were used to test for statistical indirect effects. Because expanded and traditional ACEs were highly correlated and expanded ACEs are inclusive of more forms of adversity that capture the experiences of broader sociodemographic groups (44), models were run using expanded ACEs only. It was predicted that parent *difficulties* in ER and parent ERSBs would statistically mediate the association between parent ACEs and child ER. Neither the pathway including *emotion-avoiding* ERSBs (ab = −0.0002, CI_95_ = −0.0011 to 0.0003; hypothesis 1a) nor the pathway including *emotion-accepting* ERSBs (ab = 0.0015, CI_95_ = −0.0003 to 0.0043; hypothesis 1b) was supported.

### Simple statistical mediation analyses

A secondary aim of the present project was to evaluate the simple statistical indirect effects of parenting factors (ER and ERSBs) on relations among key study variables.

**Figure 2:**
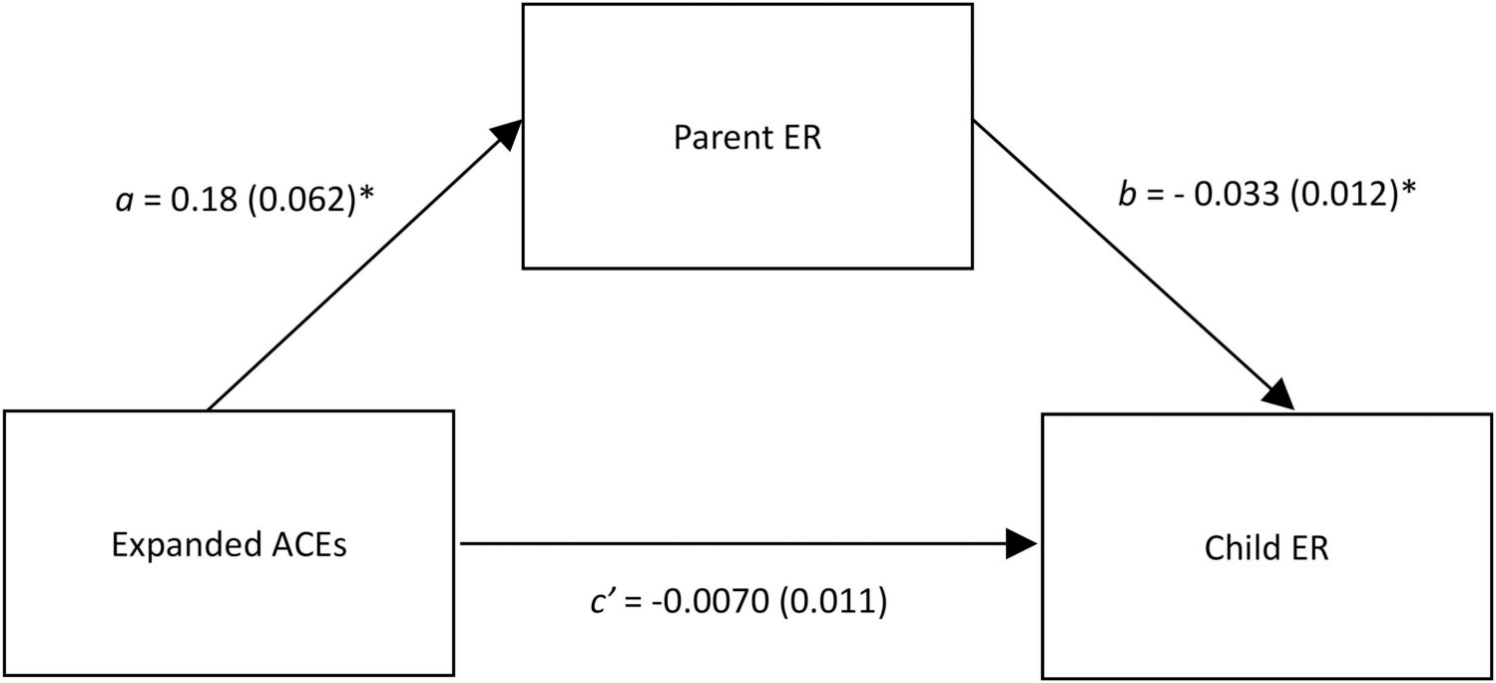
Parent emotion regulation statistically mediates the relation between expanded ACEs and child emotion regulation. This figure shows unstandardized regression coefficients. ACEs, adverse childhood experiences; ER, emotion regulation. *p < .01.

Support for statistical indirect effects was found for one hypothesized statistical mediation model (Hypothesis 2). Specifically, it was found that parent *difficulties* in ER statistically mediated the association between parent ACEs and child ER. Unstandardized path coefficients and standard errors for this model are presented in Figure 2. It was found that a higher expanded ACEs score was associated with more parent difficulties in ER, *a* = .18, CI_95_ = (0.056–0.30), and these difficulties were related to lower child ER, *b* = −0.033, CI_95_ = (−0.057 to −0.0089). The effect of ACEs on child ER was significantly statistically mediated by parent difficulties with ER, ab = −0.0060, CI_95_ = −0.0122 to −0.0013.

No support for statistical indirect effects of parent ACEs on child ER via either emotion-avoiding (ab = 0.0009, CI_95_ = −0.0018 to 0.0053; hypothesis 3a) or emotion-accepting (ab = 0.0028, CI_95_ = −0.0054 to 0.011; hypothesis 3b) ERSBs was found. Likewise, separate tests examining both emotion-avoiding (ab = 0.0050, CI_95_ = −0.0077 to 0.021; hypothesis 4a) and emotion-accepting ERSBs (ab = 0.013, CI_95_ = −0.0030 to 0.035; hypothesis 4b) as an outcome variable revealed no significant statistical indirect effects of parent ACEs on parent ERSBs via parent ER difficulties. Similarly, regardless of whether emotion-avoiding (ab = −0.0007, CI_95_ = −0.0045 to 0.0018; hypothesis 5a) or emotion-accepting (ab = 0.0085, CI_95_ = −0.0012 to 0.02; hypothesis 5b) ERSBs were tested as mediators, no significant statistical indirect effects of parent ER difficulties on child ER via parent ERSBs were detected. Unstandardized beta coefficients for all hypothesized paths are presented in Table 3.

**Table 3:** Unstandardized regression coefficients for mediation analyses.

Effect	Unstandardized beta coefficients *b* (95% CI)
Hypothesis 1a	
Indirect effect of parent ACEs on child ER via parent ER and Av-ERSBs	−0.0002 (−0.0011 to 0.0003)
Hypothesis 1b	
Indirect effect of parent ACEs on child ER via parent ER and Ac-ERSBs	0.0015 (−0.0003 to 0.0043)
Hypothesis 2	
Direct effect of ACEs on parent ER	0.18* (0.056 to 0.30)
Direct effect of ACEs on child ER	−.0070 (−0.030 to0.015)
Direct effect of parent ER on child ER	−0.033* (−0.057 to −0.0089)
Total effect of ACEs on child ER	−0.013 (−0.034 to 0.0090)
Indirect effect of ACEs on child ER via parent ER	−0.0060* (−0.012 to −0.0013)
Hypothesis 3a	
Indirect effect of ACEs on child ER via Av-ERSBs	0.0009 (−0.0018 to 0.0053)
Hypothesis 3b	
Indirect effect of ACEs on child ER via Ac-ERSBs	0.0028 (−0.0054 to 0.011)
Hypothesis 4a	
Indirect effect of parent ACEs on Av-ERSBs via parent ER	0.0050 (−0.0077 to 0.021)
Hypothesis 4b	
Indirect effect of parent ACEs on Ac-ERSBs via parent ER	0.013 (−0.0030 to 0.035)
Hypothesis 5a	
Indirect effect of parent ER on child ER via Av-ERSBs	−0.0007 (−0.0045 to 0.0018)
Hypothesis 5b	
Indirect effect of parent ER on child ER via Ac-ERSBs	0.0085 (−0.0012 to 0.020)
*Post hoc* analyses	
Indirect effect of parent ACEs on child ER via parent ER and problem-focused ERSBs	0.0007 (−0.0005 to 0.0023)
Indirect effect of parent ACEs on child lability/negativity via parent ER and distress ERSBs	0.0005 (−0.0004 to 0.0020)

ACEs, adverse childhood experiences; ER, emotion regulation; Av-ERSBs, emotion-avoiding emotion-related socialization behaviors; Ac-ERSBs, emotion-accepting emotion-related socialization behaviors.

* *p* < .05.

### *Post hoc* analysis

Given the dearth of significant findings related to ERSBs, exploratory *post hoc* analyses were run to better understand associations between ERSBs and ACES, parent ER, and child ER.

First, multiple regression analyses were run to examine whether parent ER and ERSBs uniquely and independently contribute to child ER in the proposed models. The overall regression model incorporating *emotion-accepting* ERSBs was significant, *F*_(6, 207)_ = 10.03, *p* < .01 and explained 22.5% of the variance in child ER. Parent *difficulties* with ER were uniquely associated with lower child ER (*b* = −0.04, *p* < .01), while *emotion-accepting* ERSBs were uniquely associated with better child ER (*b* = 0.11, *p* < .01) when controlling for other study variables and covariates.

When *emotion-avoiding* ERSBs were tested in the model, the model remained significant, *F*_(6, 207)_ = 2.75, *p* = .01 but explained only 7.4% of the variance in child ER. Parent ER difficulties similarly were associated with lower child ER (*b* = −0.03, *p* < .01), while *emotion-avoiding* ERSBs did not contribute significantly to the model (*b* = −0.03, *p* = .15).

Because it is possible that individual aspects of emotion-accepting or emotion-avoiding ERSBs may vary in terms of how affected they are by a parent's trauma history or ER difficulties, as well as their relative associations with children's ER development, bivariate correlations between individual CTNES subscales that comprise both emotion-accepting and emotion-avoiding ERSBs and other key study variables were run to help tease apart their significance. CTNES subscales that correlated most highly with other key study variables included problem-focused reactions, which was correlated with the emotion regulation subscale of the ERC (*r* = .48, *p* < .01), and the distress reactions subscale, which was not a part of either composite scale, correlated with parent difficulties in ER (*r* = .38, *p* < .01), as well as the lability/negativity subscale of the ERC (*r* = .34, *p* < .01).

The isolation of these subscales did not uncover any additional statistical indirect effects. When the problem-focused reactions subscale was inserted into the multiple statistical mediation model as a proxy for *emotion-accepting* ERSBs, and the emotion regulation subscale of the ERC was used as the outcome variable, statistical indirect effects remained nonsignificant (ab = 0.0007, CI_95_ = −0.0005 to 0.0023). Likewise, when the distress reactions subscale was inserted into the multiple statistical mediation model as a proxy for *emotion-avoiding* ERSBs, and the lability/negativity subscale of the ERC was used as the outcome variable, statistical indirect effects were not significant (ab = 0.0005, CI_95_ = −0.0004 to 0.0020).

## Discussion

Research examining the intergenerational transmission of ACEs demonstrates that pathways that influence the likelihood of adaptive vs. maladaptive outcomes in young children are often set into motion long before their birth. Because emotion dysregulation is a transdiagnostic construct that is associated with a broad range of internalizing and externalizing psychopathology (53, 54), it is valuable to examine constructs that covary with the development of adaptive ER in young children. The present study investigated whether parent ER and ERSBs could statistically mediate the intergenerational effects of ACEs on children's ER.

### Relations between emotion-related parenting factors and child ER

Many of the findings of prior literature were supported by the present analyses. Indeed, support was found for a small negative correlation between parent difficulties in ER and child ER (24–28) and a medium positive correlation between *emotion-accepting* ERSBs and child ER (14, 29). Additionally, although it was hypothesized that parent difficulties in ER would be associated with fewer *emotion-accepting* ERSBs based on theoretical connections between these constructs, the finding that parent difficulty with ER was not associated with parent *emotion-accepting* ERSBs in the present study is consistent with previous literature that failed to find a statistical assocation between these constructs (26).

However, the present study failed to replicate past findings linking parents' *emotion-avoiding* ERSBs with child ER (14, 29), and parent ER difficulties with parent *emotion-avoiding* ERSBs (26, 31).

### Associations between parent ACEs and emotion-related parenting factors

While past studies have shown that parent ACEs and other measures of early adversity are associated with worse ER outcomes among children (32, 33), the present study found only a small, non-significant negative correlation between expanded ACEs and child ER. Past literature examining these relations has included both behavioral observations and biomarkers of child ER rather than parent-reported ER. As such, difficulty replicating these findings could be partially accounted for by methodological limitations associated with the exclusive use of self-report data. It is also worth noting that these relations are probabilistic and not deterministic; many families are resilient, and consistent with the principle of multifinality, intergenerational transmission of ACEs is not a given, nor is it a given that exposure to adversity results in trauma. Nonetheless, findings from the present study suggested that these constructs do share an indirect statistical relation via parent ER, to be discussed in greater detail below.

The present study successfully replicated past findings regarding associations between parent ACEs and parent ER, contributing to the well-established evidence base that ACEs are positively associated with difficulties in ER (37, 38).

To our knowledge, the present study was the first to examine the relation between parent ACEs and ERSBs. Past studies have shown that a parent's maltreatment history—a related construct—is associated with less use of *emotion-accepting* ERSBs, though findings regarding associations between maltreatment history and *emotion-avoiding* ERSBs have been mixed (35, 41, 42). In contrast, the present study found no association between ACEs and emotion-accepting ERSBs, and a small, nonsignificant negative correlation between traditional ACEs and *emotion-avoiding* ERSBs. These inconsistent findings further underscore the importance of studying these relations and are discussed in further detail below.

### Pathways between parent ACEs and child ER

Results of the present study suggest that parental ER may represent an important target for interventions aiming to disrupt the intergenerational transmission of ACEs. Although prior studies have linked experiences of early adversity with emotion regulation difficulties in adulthood and cross-generationally, and a wide literature base supports the presence of a positive association between parent and child ER, the present study is the first to our knowledge that indicates that parent ER difficulties statistically mediate the association between parent ACEs and poorer child ER. Specifically, parents who endorsed experiencing more adversity during childhood reported greater difficulty with ER, which was associated with worse ER outcomes in their own children when controlling for parent age, gender, and subjective SES.

Data suggested that parent *emotion-accepting* ERSBs were uniquely associated with child ER even when accounting for the effects of parent ER, SES, gender, and age. However, there was no evidence that parent ERSBs statistically mediate either the relation between parent ACEs and child ER or the relation between parent ER and child ER. These findings suggest that, although related to child ER, parent ERSBs may represent a less appropriate target for interventions whose goal is specifically to interrupt the intergenerational transmission of parent ACEs in parents with young children. Prior literature examining this pathway has been inconsistent (24, 26).

Despite results not supporting the hypothesized statistical indirect effects of ERSBs, the fact that ERSBs failed to statistically mediate associations between key constructs while ER did not is not altogether surprising. Emotion regulatory capacities can be directly affected by trauma—indeed, several symptoms necessary for meeting criteria for posttraumatic stress disorder feature, at their core, difficulties regulating emotions. Parents' emotion regulatory capacity may in turn have significant bearing on all of the factors hypothesized to influence the development of children's ER in Morris' Tripartite Model (23). In contrast, parent ERSBs, while still hypothesized to play a role, represent specific, potentially learned practices that are likely influenced by a broad host of factors that may include but are certainly not limited to parents' own ER and history.

Of note, many interventions targeting the cultivation of ER skills in young children have been developed with a primary focus on improving parents' ERSBs. A review of these interventions reveals that they vary in terms of their attention to parent ER (55). Interestingly, while evaluations of these interventions have demonstrated improvements in targeted ERSBs and related beliefs, the majority of these studies have failed to find support for treatment effects on child ER or did not measure child ER (55). Other interventions, including an emotion coaching intervention for families exposed to intimate partner violence, have placed a greater emphasis on parental ER (56). In contrast to interventions that lack such an emphasis, participation in this program was associated with improvements in children's ER in addition to parenting practices (56), providing additional and practical support that parent ER may be an important avenue through which to disrupt the intergenerational transmission of early adversity.

### Is there a relation between parental emotion regulation and parental behavior?

It is noteworthy that parent ER difficulties were not found to be associated with either emotion-accepting or emotion-avoiding ERSBs in the present study despite the presence of both theoretical and empirical support for associations between these constructs (22, 26, 31). Overall inconsistent findings in studies examining ERSBs suggest that strong correlates of ERSBs have not yet emerged in the literature, underscoring the importance of further research in this area. Although it is certainly possible that these constructs were not related in the current sample because other factors were simply more likely to covary with ERSBs than ER, it is worth considering whether social desirability bias could have played a role. As noted, the CTNES was adapted from a similar measure (i.e., the Coping with *Children's* Negative Emotions Scale; 50) whose psychometric properties and robustness against social desirability bias have been established (57). However, the adapted version utilized in the present study was developed more recently and has been less widely used. Potential explanations for the null findings within the present study include the possibility that parents may be more susceptible to social desirability bias when reporting on emotion-related parenting with respect to younger children, or that other variables could moderate this relation. These possibilities should be explored in future work.

### Strengths, limitations, and future directions

Strengths of the present study include features of the study sample (i.e., including fathers and individuals reporting lower socioeconomic standing), the specific statistical mediators under study, and the use of a more inclusive measure of early adversity. In contrast to prior literature, these considerations help to expand our understanding of the relations between these constructs, including candidate mechanisms for future study, among broader and more diverse populations of parents.

Prior parenting literature largely focuses on *maternal* ACEs history, with limited studies including fathers (1, 34). However, it is important to understand whether, and if so, how, relations between these constructs are consistent or vary by parent gender, particularly as norms regarding family composition and traditional gender roles shift. In the present study, parental ERSBs did differ significantly by gender, which has been reported in other studies (58). Although the present study was underpowered to analyze statistical mediational relationships by gender, the inclusion of male and female parents enables preliminary analyses of various associations that may have important implications for future research and intervention.

Furthermore, while the negative intergenerational impacts of parental (and particularly maternal) ACEs have been well-documented, this fact alone has limited implications for intervention/prevention. The ability to meaningfully alter these trajectories depends on studying mechanisms through which adversity may be passed down intergenerationally, which involves building evidence both for *and against* hypothesized mechanisms. Although methodological considerations of the present study preclude inferences regarding causality (discussed in greater detail below), this study contributes data that provide additional insights into possible mechanisms that may inspire more rigorous research to continue investigating these pathways and ultimately inform intervention approaches.

An additional strength of the present study includes its use of a more inclusive measure of early adversity compared to past research, which has focused on the intergenerational effects of childhood maltreatment more narrowly. This is important, because past research has indicated that even traditional ACEs may not sufficiently capture the full breadth of adversity experienced by diverse populations (44), thus limiting generalizability of study findings. In this study, expanded ACEs were found to have a small, nonsignificant negative correlation with child ER, where no association was found between traditional ACEs and child ER. Although one ought to be skeptical about the true nature of this distinction due to the small magnitude and lack of statistical significance at the *p* < .05 level, this finding suggests that using a broader measure of adversity may more accurately capture the intergenerational effects of trauma and that adversity that occurs outside of the household is also associated with intergenerational effects, meriting further exploration in future studies.

Although this study lays important groundwork for future, more rigorous evaluation of the relations between studied constructs, critical methodological limitations should be held in mind. For instance, the present study was cross-sectional in nature, with all data having been collected at a single timepoint. As such, this study assesses for statistical indirect effects only, and no inferences can be made regarding causality or directionality. Thus, although data provide support for the notion that parent ER may be an important target for interrupting the intergenerational transmission of early adversity, replicating these findings using a longitudinal dataset would lend more support for this assertion and is an important next step and necessary for inferring causality. Of note, 11 of 41 studies included in a recent systematic review and meta-analysis examining mediators and moderators in the relationship between maternal early adversity and children's outcomes were cross-sectional in design, highlighting that this may be an important future direction for the field as a whole.

Additionally, the use of exclusively self-report data in the present study represents a meaningful limitation that further demands caution in interpreting findings. Despite the fact that self-report data are widely relied upon in clinical settings and social sciences research, these data are susceptible to bias (e.g., social-desirability bias) that can lead to both over- and under-reporting compared to “true” or more objectively measured values (59). Furthermore, the present data were collected in the context of an ongoing global pandemic. Past research has identified a broad range of factors that may bias recall of early experiences of adversity, including concurrent mental health, psychological distress, and chronic stress (60). Given that parent ACEs were assessed retrospectively during a pandemic and from parents reporting increased socioeconomic stress, these factors could have led to over-reporting of ACEs. Concerns regarding the validity of self-report data underscore the importance of using a multimethod approach to data collection. For example, the inclusion of physiological measures of emotion regulation and behavioral observations of parenting practices and child ER would provide data that are less susceptible to these forms of bias and improve confidence in study findings. Future studies should use a multimethod approach and include embedded measures of social desirability bias to better understand its effects in parent self-report of behavior across child age and parent gender.

Despite efforts to increase the generalizability of findings by including fathers and broadening the nature of adversity studied, the present sample is still relatively homogeneous in terms of race, with the majority (i.e., over 80%) of parents identifying as White. Additionally, transgender and gender nonconforming parents are poorly represented in the present study. Future research would benefit from recruitment strategies that prioritize the inclusion of diverse participants in terms of race, ethnicity, and gender identity.

Taken together, results suggest that enhancing a parent's ability to regulate their own emotions may be an important avenue through which to disrupt the intergenerational transmission of early adversity and to improve ER outcomes among children. Furthermore, screening for parental ER difficulties may assist with the identification of families most in need of intervention. Programs emphasizing parental ER have the capacity to be impactful both as primary and secondary intervention. That is—not only could this approach prevent the initiation of maladaptive pathways in children, but it also may provide an opportunity to alter the developmental trajectory of parents whose pathways have been adversely affected by early life stress.

## Data Availability

The raw data supporting the conclusions of this article will be made available by the authors, without undue reservation.
